# Protocol for an economic evaluation of scalable strategies to improve mental health among perinatal women: non-specialist care delivered via telemedicine vs. specialist care delivered in-person

**DOI:** 10.1186/s12888-023-05318-2

**Published:** 2023-11-08

**Authors:** Daisy R. Singla, Claire de Oliveira, Sean M. Murphy, Vikram Patel, Jaime Charlebois, Wendy N. Davis, Cindy-Lee Dennis, J. Jo Kim, Paul Kurdyak, Andrea Lawson, Samantha Meltzer-Brody, Benoit H. Mulsant, Nour Schoueri-Mychasiw, Richard K. Silver, Dana Tschritter, Simone N. Vigod, Sarah Byford

**Affiliations:** 1 Institute for Mental Health Policy Research, Campbell Family Mental Health Research Institute, Centre for Addiction and Mental Health, Toronto, Canada; 2 Department of Psychiatry, Temerty Faculty of Medicine, University of Toronto, Toronto, Canada; 3 Lunenfeld-Tanenbaum Research Institute, Sinai Health, Toronto, Canada; 4 Institute of Health Policy, Management and Evaluation, Dalla Lana School of Public Health, University of Toronto, Toronto, Canada; 5 ICES, Toronto, Canada; 6 Department of Population Health Sciences, Weill Cornell Medical College, New York, NY USA; 7 Department of Global Health and Social Medicine, Harvard Medical School, Boston, USA; 8 Department of Global Health and Population, Harvard Chan School of Public Health, Boston, USA; 9 Canadian Perinatal Mental Health Collaborative, Barrie, Canada; 10 Postpartum Support International, Portland, USA; 11 Lawrence S. Bloomberg Faculty of Nursing, University of Toronto, Toronto, Canada; 12 Department of Obstetrics & Gynecology, NorthShore University HealthSystem, Evanston, USA; 13 Department of Psychiatry, School of Medicine, University of North Carolina, Chapel Hill, USA; 14 Department of Obstetrics and Gynecology, University of Chicago Pritzker School of Medicine, Chicago, USA; 15 Women’s College Hospital, Toronto, Canada; 16 King’s Health Economics, Institute of Psychiatry, Psychology & Neuroscience, King’s College London, London, UK

**Keywords:** Economic evaluation, Protocol, Perinatal mental health, Randomized controlled trial

## Abstract

**Background:**

Perinatal depression affects an estimated 1 in 5 women in North America during the perinatal period, with annualized lifetime costs estimated at $20.6 billion CAD in Canada and over $45.9 billion USD in the US. Access to psychological treatments remains limited for most perinatal women suffering from depression and anxiety. Some barriers to effective care can be addressed through task-sharing to non-specialist providers and through telemedicine platforms. The cost-effectiveness of these strategies compared to traditional specialist and in-person models remains unknown. This protocol describes an economic evaluation of non-specialist providers and telemedicine, in comparison to specialist providers and in-person sessions within the ongoing Scaling Up Maternal Mental healthcare by Increasing access to Treatment (SUMMIT) trial.

**Methods:**

The economic evaluation will be undertaken alongside the SUMMIT trial. SUMMIT is a pragmatic, randomized, non-inferiority trial across five North American study sites (*N* = 1,226) of the comparable effectiveness of two types of providers (specialist vs. non-specialist) and delivery modes (telemedicine vs. in-person) of a behavioural activation treatment for perinatal depressive and anxiety symptoms. The primary economic evaluation will be a cost-utility analysis. The outcome will be the incremental cost-effectiveness ratio, which will be expressed as the additional cost required to achieve an additional quality-adjusted life-year, as assessed by the EuroQol 5-Dimension 5-Level instrument. A secondary cost-effectiveness analysis will use participants’ depressive symptom scores. A micro-costing analysis will be conducted to estimate the resources/costs required to implement and sustain the interventions; healthcare resource utilization will be captured via self-report. Data will be pooled and analysed using uniform price and utility weights to determine cost-utility across all trial sites. Secondary country-specific cost-utility and cost-effectiveness analyses will also be completed. Sensitivity analyses will be conducted, and cost-effectiveness acceptability-curves will be generated, in all instances.

**Discussion:**

Results of this study are expected to inform key decisions related to dissemination and scale up of evidence-based psychological interventions in Canada, the US, and possibly worldwide. There is potential impact on real-world practice by informing decision makers of the long-term savings to the larger healthcare setting in services to support perinatal women with common mental health conditions.

**Supplementary Information:**

The online version contains supplementary material available at 10.1186/s12888-023-05318-2.

## Background

Depression is the leading cause of disability among perinatal (i.e., pregnant and up to 1 year postpartum) women worldwide [1], affecting an estimated 1 in 5 women during the perinatal period in Canada and the United States (US) [2–4]. In this study, we define perinatal women as all perinatal persons regardless of their gender identity or expression and will refer to the sample as ‘women’ hereafter. Based on estimates from the United Kingdom (UK) [5], the annualized lifetime costs of perinatal depression may be as high as $20.6 billion CAD in Canada and over $45.9 billion USD in the US. Psychological treatments, such as cognitive, behavioural and interpersonal therapies, have consistently been shown to be effective in preventing and treating perinatal depression and anxiety [6, 7] and are generally preferred over medication as a first line treatment by perinatal women [8, 9]. Unfortunately, access to psychological care remains limited for most perinatal women with depression or anxiety [10]. Some barriers to effective mental health care can be addressed through task-sharing—the rationale distribution of tasks to non-specialist providers [11, 12] (i.e., individuals with no formal training or degree in mental health care [13]) and through telemedicine platforms, which offer an alternative approach for perinatal women in terms of flexibility [14], efficiency [15], and cost [16]. However, the cost-effectiveness of these more scalable approaches compared to traditional specialist and in-person models remains unknown. This is a significant gap that must be addressed to inform policy and wide scale uptake of these approaches.

### Economic evidence of psychological treatments

Globally, investing in psychological treatments for depression and anxiety has been shown to provide a substantial return on investment. Benefit-to-cost ratios of 2.3–3.0 to 1 have been shown when considering only the economic benefits of enhanced labor productivity, and substantially more (3.3–5.7 to 1) when the intrinsic value of improved health returns is also included [17]. Increasingly, studies are also examining the cost-effectiveness of telemedicine platforms to deliver psychological treatments. For example, among outpatients with major depressive disorder, computer-assisted forms of cognitive behaviour therapy were highly cost-effective compared to conventional therapy and reduced the adjusted cost of treatment by $945 USD per patient [18]. Another study among veterans receiving behavioural activation found that, although intervention costs for telehealth were higher relative to in-person care, veterans receiving behavioural activation via telehealth had lower health costs one year after the intervention than those receiving care in person [19]. Recent research from the UK predicted that internet-delivered behavioural therapy may achieve equivalent wellbeing outcomes with notably lower costs compared to in-person behavioural therapy over a two year horizon [20].

In terms of perinatal mental health, a recent review on the cost-effectiveness of psychological interventions for perinatal populations identified only eight studies that targeted perinatal depression or anxiety symptoms, with inconclusive results and few comparisons of active treatments [21]. Two interventions were found to be likely cost-effective: (i) screening and talk therapy (i.e., cognitive behaviour approach or person-centered approach) delivered by a health visitor [22] (i.e., a community health care worker focusing on perinatal populations); and (ii) psychiatrist-supported general practitioner screening and treatment of postpartum depression and psychosis [23]. Despite the widespread effectiveness of psychological treatments for perinatal populations [6, 7, 24], there remains a paucity of evidence on the cost-effectiveness of psychological treatments for perinatal depression and anxiety. This research is essential to inform the translation of research into practice and policy. Moreover, this information can serve as an important input to guide decisions around resource allocation.

### Objective

The objective of the proposed economic evaluation is to assess the cost-effectiveness of provider type – non-specialist (e.g., nurses or midwives) vs. specialist providers (e.g., psychiatrists, psychologists, or social workers) – and mode of delivery – telemedicine vs. in-person sessions – within the Scaling Up Maternal Mental health care by Increasing access to Treatment (SUMMIT) trial [25]. In line with the larger SUMMIT trial, the primary outcome will be derived from data pooled across both countries (Canada and the US) using a common set of unit costs. This approach will enhance internal, and possibly external, validity [26]. Due to cross-border heterogeneity in factors such as healthcare practice and resource utilization patterns, a secondary country-specific analysis will be conducted. Outcomes from this secondary analysis will be derived independently using country-specific data [26].

## Methods

### Brief description of the intervention and comparators

SUMMIT is a large, multi-site, four-arm, randomized, non-inferiority trial for perinatal women with depressive and anxiety symptoms. It is currently being implemented in Toronto, Chicago and Chapel Hill (*N* = 1,226). The objective of the SUMMIT trial is to compare the effectiveness of provider (non-specialist vs. specialist providers) and delivery mode (telemedicine vs. in-person), implementing a brief, evidence-based behavioural activation treatment. The treatment consists of six to eight individual weekly sessions and the same treatment is provided to individuals in all four study arms – telemedicine non-specialist, telemedicine specialist, in-person non-specialist, and in-person specialist. The current treatment manual has been adapted from two, well-established source manuals: the Alma Program for perinatal populations in Colorado [27], and the Healthy Activity Program from Goa, India [28, 29]. Key treatment strategies include psychoeducation, behaviour assessment, values-based activity monitoring and structuring, interpersonal effectiveness, and problem solving. Unlike traditional cognitive behaviour interventions for depression, behavioural activation explicitly targets avoidant coping and has also been effective in reducing symptoms of anxiety [30].

The SUMMIT treatment providers are either non-specialist or specialist providers. Non-specialist providers include registered and practictioner nurses, midwives, or doulas with general or obstetric health care professional skills but without formal training in mental health care or experience delivering psychological treatments. Specialist providers include individuals with formal training in mental health care delivery (e.g., psychiatrists, psychologists, or social workers) and a minimum of 5 years of experience delivering psychological treatments [31]. Telemedicine is implemented via Zoom™ in Toronto and Chicago, and Webex™ in Chapel Hill. All platforms permit video visits; are accessible on PC, Mac, Android, and iOS systems; and are compliant with the Personal Health Information Protection Act/Health Insurance Portability and Accountability Act (PHIPA/HIPAA). Study tablets and access to high-speed Internet are provided, with instructions for use, on a temporary basis to participants lacking access to a phone, tablet, or computer. Participants assigned to telemedicine can do their behavioural activation sessions in whatever private location they prefer (e.g., home or elsewhere). In-person sessions are held at participating clinical care sites. During the COVID-19 pandemic, in-person arms were temporarily suspended to decrease the number of non-urgent visits to hospitals and clinics and to decrease the risk of exposure to COVID-19 for both study participants and treatment providers, in line with institutional guidelines. Additional details can be found elsewhere [32]. The larger SUMMIT trial has been registered on ClinicalTrial.gov, NCT 04153864.

### Setting and sample

The clinical trial is currently being conducted at three academic hospitals in Toronto, Ontario, Canada (Sinai Health, Women’s College Hospital, and St. Michael’s Hospital), and two health care institutions in the US (NorthShore University HealthSystem, Evanston, Illinois and UNC Health and UNC School of Medicine, Chapel Hill, North Carolina). A total of *N* = 1,226 pregnant and postpartum women are being recruited through an established extensive referral system at each site. This sample size was determined by the larger SUMMIT Trial [32] and detailed elsewhere (ClinicalTrials.gov NCT 04153864). The sample size calculation was based on the primary outcome Edinburgh Postnatal Depression Scale (EPDS [33]), with an EPDS mean estimate of 7.93 (SD = 4.68) [34] and powered to estimate the two primary comparisons of treatment provider type (non-specialist vs. specialist provider) and delivery mode (telemedicine vs. in-person) within a non-inferiority design. The clinical trial enrolls women with an EPDS score ≥ 10 (indicative of minor and major depression [3, 35]); ≥ 18 years of age; pregnant up to 36 weeks or 4 to 30 weeks postpartum; and who speak English (in Canada) or English or Spanish (in the US). The trial excludes women with active suicidal intent; symptoms of psychosis or mania; initiation or change in psychotropic medication or dosage within 2 weeks of enrollment; ongoing psychotherapy; active substance use or dependence; or severe fetal anomalies, stillbirth, or infant death at time of enrolment for index pregnancy. Recruitment for the larger SUMMIT trial began on January 06 2020 and data collection is ongoing.

### Decision problem

The SUMMIT trial will assess the effectiveness of provider and delivery mode in implementing a brief, evidence-based behavioural activation treatment for perinatal women with depressive and anxiety symptoms. Therefore, the proposed economic evaluation of SUMMIT will determine the relative cost-effectiveness of the four study arms created by randomizing participants to non-specialist vs. specialist providers and to telemedicine vs. in-person sessions (in line with the clinical analyses). In the secondary country-specific analysis, separate analyses will be undertaken for each country following the current guidelines most applicable to each context. The guidelines recommended by the Canadian Agency for Drugs and Technologies in Health (CADTH) [36] will be followed in Canada while the Second Panel on Cost-Effectiveness in Health and Medicine [37] and Glick et al. [26] will be followed for the American analysis. Both analyses will follow the Consolidated Health Economic Evaluation Reporting Standards (CHEERS) 2022 reporting guidance for health economic evaluations [38].

The primary economic evaluation will be a cost-utility analysis where the incremental cost-effectiveness ratio (ICER) will be expressed as the additional cost required to achieve an additional quality-adjusted life-year (QALY). This analysis will be carried out at the 3-month (intervention) and 12-month (intervention + follow-up) post-randomization assessments, based on non-missing data (i.e., excluding individuals lost to follow-up or with missing cost and/or outcome data). The potential impact of non-responders will be examined through sensitivity analyses (described below). The 12-month ICER will serve as the primary outcome of interest. A secondary analysis will include a cost-effectiveness analysis using the primary clinical outcome measure of the trial, the EPDS [33]. The advantage of undertaking this secondary analysis is the opportunity to explore cost-effectiveness using a measure that may be more sensitive to change in the population of interest. While QALYs are preferred to support comparisons across disorders and populations, their broad, generic nature can make them less sensitive to change in specific populations. While studies designed to test equivalence of effects are a legitimate situation in which a cost-minimisation analysis (i.e., an analysis where costs alone are compared given equal outcomes) may be appropriate [39], the same may not hold for trials with non-inferiority designs. Even in cases where equivalence or non-inferiority are demonstrated, exploration of the joint distribution of costs and effects in a cost-effectiveness analysis is recommended to represent uncertainty [39] and to help interpret the economic results [26, 40]. For these reasons, a cost-effectiveness analysis will be undertaken, regardless of whether non-inferiority in the primary clinical outcome is demonstrated. Cost-effectiveness will be determined using the net benefit approach [41] with reference to Bosmans’ methods for economic evaluations alongside equivalence or non-inferiority trials [40]. See Table 1 for a summary of the economic evaluation outcome measures.

**Table 1:** Economic evaluation outcome measures

Outcome	Assessment	Measurement	Economic evaluation framework
Edinburgh Postnatal Depression Scale (EPDS)	baseline, session-wise, 3-, 6-, 12-months post-randomization	perinatal depressive symptoms	cost-effectiveness analysis
EuroQol 5 Dimension 5 Level (EQ-5D-5L)	baseline, 3-, 6-, 12-months post-randomization	quality of life	cost-utility analysis

### Time horizon and discounting

The time horizon of this study will be the length of the trial, which is 12-months post-randomization. Given this time horizon, discounting will not be required.

### Measurement and evaluation of health

The primary outcome of the proposed cost-effectiveness analysis will be the QALY, a measure that combines the health-related quality of life associated with an individual’s health state and their time spent in that state. The QALY is recommended as the primary effectiveness measure in economic evaluation studies due to its ability to be compared across interventions and illnesses/disorders. Health-related quality-of-life will be measured by the EuroQol 5-Dimension 5-Level (EQ-5D-5L) instrument [42]. The EQ-5D-5L consists of five health state dimensions (mobility, self-care, usual activity, pain/discomfort, and anxiety/depression) on which the respondent must indicate their health status on one of five levels (no problems, slight problems, moderate problems, severe problems, and extreme problems). An individual is assigned to a unique health state according to their combination of answers across the five domains, and each health state is associated with a utility value that reflects society’s preference for that state. This valuation is indicated by a number typically ranging between 0 (worst imaginable condition: death) to 1 (perfect health), with standardized estimates by country. The EQ-5D-5L utility values can range from -0.285 to 1, with values below 0 representing states perceived to be worse than death. Canada- [43] and US-specific utility weights [44] will be used for the pooled and respective country-specific economic evaluations [26].

As mentioned above, a secondary cost-effectiveness analysis will be conducted using the primary clinical outcome of the SUMMIT trial, participants’ depressive symptom scores measured by the EPDS [33]. The EPDS is a short, time-efficient, internationally used, and freely-available 10-item measure, which has been validated among diverse populations across the postpartum period [45], including remotely [46]. The EPDS is also the standardized tool in Canada and the US to assess perinatal mental health issues among pregnant and postpartum individuals. An EPDS ≥ 10 cut-off has been suggested to encompass both minor and major depression [3] for antenatal, postnatal, and community-based populations with excellent sensitivity and specificity [47, 48].

### Resource use and costs

#### Intervention resource use and costs

Data on the use of the behavioural activation treatment (i.e., the number and duration of therapy contacts and with whom) will be collected from existing clinical records and study data. The use of behavioural activation will be directly costed for each arm using a standard micro-costing approach [49]. Unit costs for behavioural activation will include all hospital and employer costs (provider salaries, contributions to pensions, etc.) and appropriate overhead (capital, managerial, administrative, etc.). The cost of supervision will be included and the time each therapist spends on various direct and indirect participant-related activities (non-participant contact time including training, administration, meetings with other professionals, etc.) will be estimated using a questionnaire developed by the research team. See Table 2 for a list of intervention resources, their respective unit costs and source.

**Table 2:** Measurement and valuation of intervention costs

Category	Cost Item		Non-Specialist Provider (NSP)	Specialist Provider (SP)	COVID-19 Precautions	Online/Virtual	In-Person	Notes
Training Costs	Personnel training workshop	General staff	☒	☒	☐	☒	☒	
		NSP Time	☒	☐	☐	☒	☒	
		SP Time	☐	☒	☐	☒	☒	
		Clinical Lead time	☒	☒	☐	☒	☒	
	Competency assessment	General staff	☒	☒	☐	☒	☒	
		Clinical Lead time	☒	☒	☐	☒	☒	
	Equipment for training		☒	☒	☐	☐	☒	Flip boards, markers, pens
	Standardized Patient (contracted from outside organizations)		☒	☐	☐	☒	☒	Hired actors for therapy role-plays with new providers to assess therapeutic skills and competence
	Room rental for training		☒	☒	☐	☐	☒	
	Printing BA Manual, workbook, and value cards per provider		☒	☒	☐	☒	☒	
	Meals/refreshments		☒	☒	☐	☒	☒	
Office Equipment	Audio recorder		☒	☒	☐	☒	☒	
	Office supplies		☒	☒	☐	☐	☒	
Session & Delivery Costs	Provider Insurance		☒	☒	☐	☒	☒	
	Commute and parking compensation for NSPs		☒	☐	☐	☐	☒	
	Session safety-associated costs, e.g. Call with Psychiatrist/ER visit/Security		☒	☒	☒	☒	☒	
	Supervision attendance – Clinical Lead		☒	☒	☐	☒	☒	
	Supervision attendance – SPs		☐	☒	☐	☒	☒	
	Supervision attendance – NSPs (QSUMMIT – compensation for audio review)		☒	☐	☐	☒	☒	
Personnel costs (salaries)	Compensation for Clinical Lead		☐	☒	☐	☒	☒	Hired/billing for the intervention
	Compensation for Psychiatrists		☒	☒	☐	☒	☒	Hired/billing for the intervention
	Compensation for NSPs for session delivery; cost to trial/program		☒	☐	☐	☒	☒	Breakdown: • Salary cost to NSP and SP • Employer costs (contributions to pensions/national insurance etc.) • Plus overheads (fringe) • Time spent on face to face and non-face to face contact
	Compensation for SPs; cost to OHIP		☐	☒	☐	☒	☒	
Sensitivity analysis	Laptops (bought specifically for study)		☒	☒	☐	☒	☒	May not be used; depends if participants have their own already
	Webcams		☒	☒	☐	☒	☐	
	Headsets		☒	☒	☐	☒	☐	
	"Loaner" tablets for participants (total of 5 tablets)		☒	☒	☐	☒	☐	
Overhead costs	Management/admin		☒	☒	☐	☒	☒	
	Internet		☐	☒	☐	☒	☒	
	Office space for intervention support staff		☒	☒	☐	☐	☒	
	Clinic rooms for sessions		☐	☒	☐	☐	☒	
	Fax service (initial and end of treatment)		☒	☒	☐	☒	☒	
	Utilities		☐	☒	☐	☐	☒	
	PHIPPA/HIPPA compliant Zoom – Training		☒	☐	☒	☒	☒	Paid for by the hospital
	PHIPPA/HIPPA compliant Zoom – Session Delivery		☒	☐	☒	☒	☒	
	Masks, face shield, sanitizing equipment		☒	☒	☒	☐	☐	Paid for by the hospital
	Maintenance of office equipment		☒	☒	☐	☒	☒	
	Landlines		☒	☒	☐	☒	☒	

*BA* Behavioural Activation, *ER* Emergency Room, *NSP* Non-Specialist Provider, *OHIP* Ontario Health Insurance Plan, *SP* Specialist Provider

#### Health services utilization

The use of health services will be captured using an adapted version of the Health Services Utilization Questionnaire (HSUQ, see Appendix A) [50]. The HSUQ records the self-reported use of health care services, such as psychiatric and medical hospital inpatient stays, emergency department visits, outpatient appointments, and community health contacts at baseline and at each treatment session, 3-, 6- and 12-month follow-up, as well as outpatient prescription drugs at baseline and each session only. The HSUQ was adapted with reference to other measures used in perinatal depression populations [51–53].

#### Healthcare costs

A person-level costing method will be used to value all health care resources utilized by each participant over the course of the 12-month trial. This approach consists of multiplying unit costs for each resource by the number of resource units reported by a given participant [37, 54]. Unit costs in Canada will be obtained from the Canadian Institute for Health Information, the Ontario Health Insurance Plan fee schedule, and other sources. In the US, Medicare fee-for-service payments will be used to value health care services [55], and the US Department of Veterans Affairs Federal Supply Schedule will be used to value all medications, as per the current guidelines [37]. All costs will be adjusted to 2025 Canadian and US dollars using Statistics Canada’s Consumer Price Index for Health and Personal Care [56], and the US Bureau of Labor Statistics’ Medical Care Index [57], respectively.

### Statistical analyses

Data analysis will be done using SAS (version 9.4) and Stata (version 17.0). Participants will be analysed on an intention-to-treat basis, according to the group they were randomized to, regardless of intervention compliance. Comparisons will be made between non-specialist and specialist providers and between telemedicine and in-person sessions, in line with the clinical analyses. Costs and outcomes will be compared at 3- and 12-months and will be presented as mean values by arm with standard deviations. Mean differences and 95% confidence intervals will be obtained by non-parametric bootstrap regressions to account for the non-normal distribution that is commonly found in cost data [58].

All cost and effectiveness measures will be modeled according to the person period over the 3-month intervention phase, and the entire 12-month study period. Analyses will be conducted using multivariable generalized linear mixed models (GLMMs) [26]. The GLMM is a flexible model that allows for the assessment/choice of the most appropriate mean and variance functions to be chosen, which is especially important when analyzing costs, given the tendency for non-normal distributions. Additionally, the GLMM enables the inclusion of random effects, and uses all available data for each participant, regardless of completeness, which makes it well-suited for intent-to-treat analyses. Resources will be categorized as relevant, and individual models will be estimated for each category to predict the mean cost according to time-period and study arm. The same process will be used to predict mean health utility and EPDS values, by time-period and study arm. The statistical method of recycled predictions will be utilized to obtain the final predicted mean values, which will then be summed and assessed for statistical significance over both the 3-month intervention phase and the entire 12-month study period, except for the health utility values, which will be used to estimate QALYs gained via the area under the curve methodology. Finally, the ICER will be calculated as the incremental predicted cost of a chosen strategy relative to another, divided by the incremental predicted effectiveness of the two strategies.

#### Sensitivity analysis

To explore the potential impact of excluding non-responders, the sociodemographic and clinical characteristics of those included in the analyses and those in the full sample will be examined. Furthermore, the primary and secondary cost-effectiveness analyses will be rerun with missing total costs and outcomes imputed using model-based multiple imputation by chained equations. Five imputed data sets will be generated, and results averaged across these five iterations [59]. Linear mixed models will be used to address repeated measures. The robustness of the results will also be assessed regarding variations in the unit cost estimates, and the cost of implementing and managing the interventions. A deterministic one-way sensitivity analysis will be conducted to assess the robustness of the results to variations in unit costs, as well as the trial intervention costs, where the range for the sensitivity analysis will be obtained from 95% confidence intervals. Additionally, results from the relatively robust and efficient GLMM regression will be compared to those obtained from the relatively transparent ordinary least squares regression, as well as to the unadjusted mean values. Pattern-mixture models will be employed to detect the impact of potential outliers, deviations from distributional assumptions, and the impact of other baseline prognostic factors.

#### Uncertainty

Nonparametric bootstrapping techniques will be employed within the multivariable GLMM framework to estimate standard errors and p-values for each incremental cost (including total and individual resource categories) and effect, while adjusting for sampling uncertainty in the point estimates. Deterministic one-way sensitivity analyses will be performed to determine the level of confidence around the resulting ICERs. Based on a net benefit framework [41], uncertainty will be explored using cost-effectiveness planes and cost-effectiveness acceptability curves (CEACs). Cost-effectiveness planes illustrate the uncertainty around the estimates of costs and effects by plotting the bootstrapped cost and effects, with points in each quadrant indicating a different implication for economic evaluation [60]. CEACs are an alternative to confidence intervals around ICERs and are derived from the joint distribution of incremental costs and incremental effects (e.g., QALYs gained) using results from the aforementioned non-parametric bootstrapping of the observed data. The CEAC depicts the probability that an intervention is cost-effective compared to another for a range of values that a decision maker is willing to pay for a unit improvement in the outcome of interest [61]. The commonly used cost-effectiveness threshold for Canada is $50,000 CAD per QALY gained [36]; the recommended cost-effectiveness threshold range in the US is $100,000—$200,000 USD per QALY [37]. Coefficients of differences in net benefits between the trial arms will be obtained through a series of bootstrapped linear regressions, which will include covariates included in the main clinical analysis plus the baseline variable of interest. The resulting coefficients will then be examined to calculate the proportion of times that the intervention group had a greater net benefit than the control group for each willingness to pay value [62]. These proportions will then be plotted to generate CEACs for all cost-outcome combinations. These curves are a recommended decision-making approach to dealing with the uncertainty that exists around the estimates of expected costs and expected effects associated with the interventions under investigation and uncertainty regarding the maximum cost-effectiveness ratio that a decision-maker would consider acceptable. Additionally, CEACs allow for multiway comparisons, thus enabling the research team to explore the ‘ranking’ of the 4 arms in terms of their relative cost-effectiveness.

#### Equity

All outcomes will be weighted equally regardless of the characteristics of people receiving the interventions. However, the possibility of conducting subgroup analyses related to heterogeneity due to clinical severity and perinatal period (antenatal or postnatal) will be explored, in line with the proposed clinical analyses. Moreover, a gender-based analysis plus framework will be used to assess the potential impact of identity factors (e.g., sexual orientation, marital status, race or ethnicity, education level, employment status, and age) known to be associated with socioeconomic and financial contexts among patients and health care systems.

### Engagement approach with patients and stakeholders

The SUMMIT trial includes an extensive network of stakeholders who serve on the study’s Stakeholder Advisory Committee (SAC). The SAC includes individuals with lived experience, patient advocates and community partners, web-based organisations, clinicians from various health professions, representatives from US-based insurance companies (private third-party payers) and policy makers from study sites and across North America. The SAC was formed to inform the development, implementation, and dissemination of SUMMIT. Stakeholders advised on the initial study design and continue to actively inform trial processes and dissemination strategies through a patient-centered and pragmatic lens; for example, how to best support non-specialist providers, advocacy for patient-centered priorities and serving as patient stakeholder representatives. Stakeholders will also help to interpret the results and will play a significant role in the dissemination of the study results. Stakeholders are regularly engaged through multiple platforms to ensure that all voices are represented to inform key decision making. This includes attendance at annual stakeholder meetings, attendance at monthly investigator meetings where key trial decisions and updates are discussed, and participation in qualitative interviews. Three stakeholders have been involved specifically to inform the proposed economic evaluation from both a patient advocacy and health system perspectives in both Canada and the US. These stakeholders represent Postpartum Support International [63], the Policy Center for Maternal Mental Health [64], and the Canadian Perinatal Mental Health Collaborative [65].

## Discussion

### How the economic evaluation will support evidence-based decision making in Canada and the US

One of the goals of the SUMMIT trial is to challenge traditional models of psychotherapy delivery (in-person, delivered by a mental health specialist) from an interdisciplinary perspective. It is expected that the results of this study will have significant impact on several levels. To our knowledge, this work will represent the first economic evaluation of scalable innovations (non-specialist providers and telemedicine) relative to traditional specialist and in-person models for perinatal depression and anxiety. Furthermore, these comparisons will inform whether a stepped care model can optimize available resources. As a result, this study could transform mental health care delivery across North America and beyond. Moreover, the resulting findings should be generalizable to perinatal mental health services across North America as well as other outpatient settings because the SUMMIT trial is conducted within real-world and diverse settings in both Canada and the US.

The allocation of health care resources to maximise patient-oriented outcomes is paramount to patients, families, and the health care system. In addition to informing technical efficiency (i.e., value for money) and the impact on upcoming budgets, the economic evaluation of the SUMMIT trial can inform funding allocation decisions and dissemination and scale up of evidence-based psychological interventions in Canada and the US, and globally. The results of this study will also have the potential to directly inform future implementation and scale-up projects and foster further collaborations between policy makers, clinicians, and researchers across North America and globally.

### Limitations

The proposed economic evaluation will have some limitations. For example, the utility weights that will be used in this study are for the general Canadian and American populations rather than for perinatal women. Unfortunately, there are no specific utility weights for the latter population. Furthermore, the data on health service utilization are self-reported, and thus subject to recall bias and potentially stigma-related under-reporting bias; however, the reliability and validity of self-reported data has been well established over recall periods comparable to those in this study [66–70]. Furthermore, the study may not capture all health services used by participants (e.g., outpatient prescription drugs in the post-behavioural activation treatment phase). Nonetheless, the HSUQ captures the most relevant health services used by this patient population.

### Supplementary Information

**Additional file 1.**

## Data Availability

The results of the main trial and the economic evaluation will be submitted for publication in peer-reviewed journals. The datasets used and/or analysed during the current study may be available from the corresponding author on reasonable request.

## Abbreviations

BA: Behavioural Activation
CADTH: Canadian Agency for Drugs and Technologies in Health
CEAC: Cost-effectiveness acceptability curve
CHEERS: Consolidated Health Economic Evaluation Reporting Standards
EPDS: Edinburgh Postnatal Depression Scale
EQ-5D-5L: EuroQol 5 5 Level
GLMM: Generalized linear mixed models
HIPAA: Health Insurance Portability and Accountability Act
HSUQ: Health Services Utilization Questionnaire
ICER: Incremental cost-effectiveness ratio
NSP: Non-Specialist Provider
PHIPA: Personal Health Information Protection Act
QALY: Quality-adjusted life-year
SAC: Stakeholder advisory committee
SP: Specialist Provider
SUMMIT: Scaling Up Maternal Mental healthcare by Increasing access to Treatment Trial
UK: United Kingdom
UNC: University of North Carolina
US: United States
