# Supplementary material for: Protocol for an economic evaluation of scalable strategies to improve mental health among perinatal women: non-specialist care delivered via telemedicine vs. specialist care delivered in-person
Source: BMC Psychiatry. 2023 Nov 8;23:817. doi: 10.1186/s12888-023-05318-2 (PMC10634150; doi:10.1186/s12888-023-05318-2)
Supplement: Supplementary file 1 — Additional file 1. [file 12888_2023_5318_MOESM1_ESM.docx]

**APPENDIX A. Health service utilization measures**

**1. Health Services Utilization Questionnaire (HSUQ)**

We are very interested in the other health services that you may have received in addition to the SUMMIT treatment. Please try to remember as best as you can.

Please indicate if you are pregnant or already have had your baby. Pregnant☐ Had my baby ☐

**Services Used**

Please read a list of services that you might have used since you have known that you are pregnant (if pregnant is checked)/had your baby (if had my baby is checked)/last session (when the form is completed at Treatment 2-8) /last time you completed a questionnaire for our SUMMIT study (when the form is completed at first treatment and follow-up assessments). Please indicate yes or no for each service. For any service that you did use, you will be asked a few more details.

| **Service** |  | **How Many Times?** | **Was it beneficial?** | **For mental health?** |
| --- | --- | --- | --- | --- |
| 1. Home visit by a public health nurse | - No - Yes - Not wish to answer | _______ | - No - Yes - Mixed - Not wish to answer | - No - Yes - Partially - Not wish to answer |
| 2. Home visit by another type of nurse | - No - Yes - Not wish to answer | _______ | - No - Yes - Mixed - Not wish to answer | - No - Yes - Partially - Not wish to answer |
| 3. Telephone conversation with a public health nurse | - No - Yes - Not wish to answer | _______ | - No - Yes - Mixed - Not wish to answer | - No - Yes - Partially - Not wish to answer |
| 4. Telephone conversation with another type of nurse (i.e., aside from an IPT nurse or nurse from this study) | - No - Yes - Not wish to answer | _______ | - No - Yes - Mixed - Not wish to answer | - No - Yes - Partially - Not wish to answer |
| 5. Attended a new mother’s group/program led by a nurse | - No - Yes - Not wish to answer - Not applicable | _______ | - No - Yes - Mixed - Not wish to answer | - No - Yes - Partially - Not wish to answer |
| 6. Visit by midwife | - No - Yes - Not wish to answer | _______ | - No - Yes - Mixed - Not wish to answer | - No - Yes - Partially - Not wish to answer |
| 7. Visit to a breastfeeding clinic/ to or from a lactation consultant | - No - Yes - Not wish to answer - Not applicable | _______ | - No - Yes - Mixed - Not wish to answer | - No - Yes - Partially - Not wish to answer |
| 8. Visit to a family doctor | - No - Yes - Not wish to answer | _______ | - No - Yes - Mixed - Not wish to answer | - No - Yes - Partially - Not wish to answer |
| 9. Visit to an obstetrician | - No - Yes - Not wish to answer | _______ | - No - Yes - Mixed - Not wish to answer | - No - Yes - Partially - Not wish to answer |
| 10. Visit to a pediatrician | - No - Yes - Not wish to answer - Not applicable | _______ | - No - Yes - Mixed - Not wish to answer | - No - Yes - Partially - Not wish to answer |
| 11. Visit to a psychiatrist (other than your SUMMIT treatment provider) | - No - Yes - Not wish to answer | _______ | - No - Yes - Mixed - Not wish to answer | - No - Yes - Partially - Not wish to answer |
| 12. Visit to a psychologist or therapist (other than social worker or your SUMMIT treatment provider | - No - Yes - Not wish to answer | _______ | - No - Yes - Mixed - Not wish to answer | - No - Yes - Partially - Not wish to answer |
| 13. Visit to or from a social worker | - No - Yes - Not wish to answer | _______ | - No - Yes - Mixed - Not wish to answer | - No - Yes - Partially - Not wish to answer |
| 14. Visit to an after-hours/walk-in clinic | - No - Yes - Not wish to answer | _______ | - No - Yes - Mixed - Not wish to answer | - No - Yes - Partially - Not wish to answer |
| 15. Visit to a hospital emergency department | - No - Yes - Not wish to answer | _______ | - No - Yes - Mixed - Not wish to answer | - No - Yes - Partially - Not wish to answer |
| 16. Ambulance | - No - Yes - Not wish to answer | _______ | - No - Yes - Mixed - Not wish to answer | - No - Yes - Partially - Not wish to answer |

Notes (optional): _______

**[Asked only at 12-month time point]: Please answer the following questions related to the time period since you started the study, approximately 12 months ago.**

| 17. Since you started the study, **approximately 12 months ago**, have you stayed in a hospital overnight for mental health reasons? | - No - Yes - Not wish to answer | If yes, how many nights?  _______ |
| --- | --- | --- |
| 18. Since you started the study, **approximately 12 months ago**, have you stayed in a hospital overnight for pregnancy/birth reasons? | - No - Yes - Not wish to answer | If yes, how many nights?  _______ |
| 19. Since you started the study, **approximately 12 months ago**, have you stayed in a hospital overnight for any other reason (physical health)? | - No - Yes - Not wish to answer | If yes, how many nights?  _______ |

**2. Other health service utilization measures**

| **Category** | **Cost Item** | **Data Collection Time Points** |
| --- | --- | --- |
| Health care Costs | Current medication, change in medication and new medication. | Collected at baseline and before each treatment session. |
|  | Recommendations for referrals to MH programs for further mental health care among participants who gave consent to contact their health care provider(s). | Recommendations for referrals can occur at any point in the trial and are tracked |
| Health Benefit Plan Access and Use | 1. Are you currently enrolled in an employee private health care plan (your own, partner’s, family’s?) | Collected at baseline and 3, 6 and 12 month follow-up time points. |
|  | 1. [If Yes to 1] Does your plan include coverage for psychological services? |  |
|  | 1. [If No to 1] Do you have access to an Employee Assistance Program (through own, partner’s, or family’s employer)? |  |
|  | 1. [If Yes to 1(a) or 1(b)] Have you used this service to access psychotherapy in the last (12 months [Baseline time point] or 3 months [3 and 6 month time point] or 6 months [12 month time point]? |  |
